# Virulent Type A *Francisella tularensis* actively suppresses cytokine responses in human monocytes

**DOI:** 10.3389/fcimb.2014.00045

**Published:** 2014-04-10

**Authors:** Devyn D. Gillette, Heather M. Curry, Thomas Cremer, David Ravneberg, Kavin Fatehchand, Prexy A. Shah, Mark D. Wewers, Larry S. Schlesinger, Jonathan P. Butchar, Susheela Tridandapani, Mikhail A. Gavrilin

**Affiliations:** ^1^Integrated Biomedical Graduate Program, The Ohio State UniversityColumbus, OH, USA; ^2^Department of Microbial Infection and Immunity, The Ohio State UniversityColumbus, OH, USA; ^3^Center for Microbial Interface Biology, The Ohio State UniversityColumbus, OH, USA; ^4^Division of Pulmonary, Allergy, Critical Care and Sleep Medicine, Department of Internal Medicine, The Ohio State UniversityColumbus, OH, USA

**Keywords:** *Francisella*, monocytes, cytokines, signal transduction, innate immunity

## Abstract

**Background:** Human monocyte inflammatory responses differ between virulent and attenuated *Francisella* infection.

**Results:** A mixed infection model showed that the virulent *F. tularensis* Schu S4 can attenuate inflammatory cytokine responses to the less virulent *F. novicida* in human monocytes.

**Conclusion:** *F. tularensis* dampens inflammatory response by an active process.

**Significance:** This suppression may contribute to enhanced pathogenicity of *F. tularensis*. *Francisella tularensis* is a Gram-negative facultative bacterium that can cause the disease tularemia, even upon exposure to low numbers of bacteria. One critical characteristic of *Francisella* is its ability to dampen or subvert the host immune response. Previous work has shown that monocytes infected with highly virulent *F. tularensis* subsp. *tularensis* strain Schu S4 responded with a general pattern of quantitatively reduced pro-inflammatory signaling pathway genes and cytokine production in comparison to those infected with the less virulent related *F. novicida*. However, it has been unclear whether the virulent Schu S4 was merely evading or actively suppressing monocyte responses. By using mixed infection assays with *F. tularensis* and *F. novicida*, we show that *F. tularensis* actively suppresses monocyte pro-inflammatory responses. Additional experiments show that this suppression occurs in a dose-dependent manner and is dependent upon the viability of *F. tularensis*. Importantly, *F. tularensis* was able to suppress pro-inflammatory responses to earlier infections with *F. novicida*. These results lend support that *F. tularensis* actively dampens human monocyte responses and this likely contributes to its enhanced pathogenicity.

## Introduction

*Francisella tularensis* is the highly infectious Gram-negative causative agent of the disease tularemia (Sjostedt, 2007). *F. tularensis* has further been classified into distinct subspecies including *F. tularensis* subsp. *tularensis* (*F. tularensis*; Type A), *F. tularensis* subsp. *holarctica* (*F. holarctica*; Type B), and *F. tularensis* subsp. *novicida (F. novicida). Francisella* is especially recognized for its low infectious dose and ability to cause severe illness and death, which endorses its categorization as a Category A select agent by the CDC (Sharma et al., 2011). Of note, the most life-threatening forms of tularemia are particularly associated with Type A infections regardless of host species (Mohapatra et al., 2013). Although known to infect a range of host organisms and cell types (Rick and Wu, 2007; Hall et al., 2008), *F. tularensis* has evolved to successfully infect human monocytes/macrophages where it escapes the phagosome, replicates within the cytosol, and then lyses the cell before beginning a new reinfection cycle (Gavrilin et al., 2006; Clemens and Horwitz, 2007; Jones et al., 2012; Celli and Zahrt, 2013).

One critical characteristic of *F. tularensis* is its ability to attenuate the host inflammatory immune response. Indeed, early studies in humans showed that *Francisella*-infected individuals exhibited diminished cytokine responses to endotoxin (Greisman et al., 1963). In the murine model, *F. tularensis* infection does not lead to a classic pro-inflammatory cytokine response, which in turn results in insufficient numbers of immune cells recruited to infection sites (Bosio et al., 2007). Rarther, murine studies have corroborated the findings of *Griesman et al*. (Greisman et al., 1963), where challenge with LPS after infection does not lead to the production of pro-inflammatory cytokines such as TNF-α in mouse cell lines or *in vivo* (Telepnev et al., 2003, 2005; Bosio et al., 2007). Similar findings have also been observed in *F. tularensis* infected murine bone marrow and alveolar macrophages following Pam3CSK administration (Crane et al., 2013a).

It has been shown at the cellular level that dendritic cells infected with *F. tularensis* respond poorly, exhibiting decreased cytokine production (Bauler et al., 2011). *Francisella* does not replicate within endothelial cells, nonetheless during their brief interactions (Forestal et al., 2007), *Francisella* suppresses CCL2 and CXCL8 production thus limiting pro-inflammatory activation of effector immune cells (Bublitz et al., 2010). Multiple investigations, including studies from our group, document that *F. tularensis* infected cells have a stunted and/or delayed pro-inflammatory cytokine response in contrast to other immune stimulating agents (Telepnev et al., 2003; Sjostedt, 2006; Butchar et al., 2008; Mares et al., 2008; Bosio, 2011; Dai et al., 2013). *Francisella*'s ability to dampen immune response is not only limited to single cell populations, but is also evident in multiple cell environments (Kim et al., 2008). It has been shown in murine models that *F. tularensis* Schu S4 infections are associated with a weak induction of immune related genes and an overall lack of cell infiltration within the lung, which is in contrast to what is observed in *F. tularensis* LVS, *L. pneumophila*, *P. aeruginosa* or *Y. pestis* infection (Walters et al., 2013). Concurrently, the respiratory model of tularemia is characterized by the development of tolerogenic dendritic cells, release of anti-inflammatory cytokines in the lungs and stimulation of Treg and Th17 cells (Woolard et al., 2008; Periasamy et al., 2011).

We chose to examine human peripheral blood monocytes, because a higher percentage of monocytes are infected by *F. tularensis* than either *F. holarctica* or *F. novicida* during the course of infection (Hall et al., 2008). It is well documented that avirulent *F. novicida* is capable of inducing a potent inflammatory program (Rick and Wu, 2007; Sjostedt, 2007; Cremer et al., 2009; Dai et al., 2013). In human monocytes, the focus of this study, we have previously shown that infection with *F. tularensis*, leads to diminished responses of cytokines such as TNF-α, IL-6, IL-8, and IL-12 among others (Butchar et al., 2008). Infection with *F. tularensis* also leads to the downregulation of critical host response pathway members such as TLR2, MyD88, the PI3K regulatory subunit, Type I/Type II Interferon pathway components, and factors related to autophagy (Butchar et al., 2008; Cremer et al., 2011).

The precise mechanism(s) by which the virulent *F. tularensis* can elicit dampened immune responses upon infection is still not completely understood (Oyston et al., 2004; Bosio, 2011). There is strong evidence suggesting that this bacterium can evade many of the host detection mechanisms, which leads to suboptimal immune responses and permits bacterial growth. In addition, some studies have suggested that active mechanisms are also at play, wherein *Francisella* not only escapes detection but also preemptively dampens host cell responses. For example, it has recently been shown that the lipids of *F. tularensis* but not those of *F. novicida* were capable of dampening responses to subsequent innate immune stimuli both *in vitro* and *in vivo* (Crane et al., 2013b; Ireland et al., 2013) and that interaction between C3-opsonized *F. tularensis* and Complement Receptor 3 led to host cell immunosuppression (Dai et al., 2013).

However, differentiating between active suppression by *Francisella* and the more general phenomenon of endotoxin tolerance/cross-tolerance (Greisman and Hornick, 1975; West and Heagy, 2002; Morris and Li, 2012) has not been straightforward. Tolerance consists of both an early and late phase, depends on mediators such as the inositol phosphatase SHIP (Sly et al., 2004), and can be abrogated via molecules such as IFNγ (Chen and Ivashkiv, 2010). Interestingly, IFNγ has been shown to be important for cellular resistance against *F. tularensis* in both human and mouse macrophages (Edwards et al., 2010).

Using a mixed infection model, we show that the virulent *F. tularensis* Schu S4 can attenuate pro-inflammatory cytokine responses to the less virulent *F. novicida* in human monocytes. This process is dose-dependent and requires that *F. tularensis* is viable. Importantly, our results show that *F. tularensis* can dampen monocyte responses that are already in progress, suggesting that it is bacterially-driven suppression rather than host-cell-mediated tolerance. These results indicate that although *F. tularensis* may evade detection by host innate immune sensors, it also actively antagonizes host cell responses.

## Materials and methods

### Human peripheral blood monocyte isolation

Human Peripheral blood monocytes (PBM) were isolated as previously described (Butchar et al., 2008) using centrifugation through a Ficoll gradient followed by CD14-positive selection by Magnet-Assisted Cell Sorting (MACS, Miltenyi Biotec, Auburn, CA). This results in a ≥98% pure CD14-positive population of monocytes that has been confirmed by flow cytometry. Cells were incubated at 37°C with 5% CO_2_ supplementation.

### Bacterial strains and monocyte infections

All infections were performed with PBM in antibiotic-free RPMI-1640 media (Gibco-BRL, Rockville, MD) supplemented with 10% heat-inactivated fetal bovine serum (FBS, Hyclone, Logan, UT) and 1% L-glutamine (Invitrogen, Carlsbad, CA). *F. novicida* - U112 (JSG1819) and *F. tularensis* (Schu S4) were generously provided by Dr. John Gunn (The Ohio State University, Columbus, OH) and grown on Chocolate II agar plates (Becton, Dickinson and Company, Sparks, MD) at 37°C. All experiments involving *F. tularensis* were performed in a BSL3 facility at The Ohio State University as previously described (Butchar et al., 2007). Bacteria were resuspended in RPMI-1640 and then quantified by a spectrometer at 600 nm wave-length to calculate Multiplicity of Infection (MOI). Heat-killed *F. tularensis* (t°) was prepared by heating at 95°C for 10 min. Paraformaldehyde (pf) -killed *F. tularensis* was prepared by treating with 4% paraformaldehyde for 40 min, followed by five washes in PBS and two washes in RPMI to quench residual aldehydes as previously described (Gavrilin et al., 2006; Cremer et al., 2012). Treated bacterial suspensions were plated on chocolate II agar plates to ensure effective killing.

For infection, monocytes were resuspended in polypropylene tubes (Fisher Scientific) in RPMI medium 1640 supplemented with 10% FBS (endotoxin-free; HyClone), at 1 or 2 × 10^6^ cells per tube. Live or killed bacteria were added to cells individually or together at various multiplicities of infection (MOI), specified for every experiment. Cells were harvested 2, 4, 16-18, and 24 h after infection; separated from bacteria by low-speed centrifugation at 1000 g for 5 min; and lysed in TRIzol® (Invitrogen, Carlsbad, CA) or hypotonic lysis buffer for RNA or protein isolation, respectively. After low-speed centrifugation, cell culture media was cleared from bacteria by high speed centrifugation at 16,000 g for 5 min, filtered and used for cytokine determination.

### Real-time RT-qPCR

Quantitative Reverse-Transcription PCR was performed in detail as described previously (Gavrilin et al., 2006). In short, RNA was extracted from human PBM using TRIzol® reagent (Invitrogen, Carlsbad, CA), reverse transcribed to cDNA, and then amplified using SYBR Green PCR master mix (Eurogentec North America, San Diego, Ca). Real-time PCR was performed on an Applied Biosystems StepOne Plus system, with automatically-calculated thresholds. Primer sequences used to amplify cDNA are as follows: *IL6* (forward, 5′-CACAGACAGCCACTCACCTC-3′; reverse, 5′-TTTTCTGCC AGTGCCTCTTT-3′), *IL8* (forward, 5′-AGTTTTTGAAGAGGGCTGAGAAT-3′; reverse, 5′-CAACAGACCCACACAATACATGA-3′), and *TNF* (forward, 5′-GCTTGTTCCTCAGCC TCTTCT-3′; reverse, 5′-GGTTTGCTACAACATGGGCTA-3′). The housekeeping gene sequences are *CAP1* (forward, 5′-ATTCCCTGGATTGTGAAATAGTC-3′; reverse, 5′-ATT AAAGTCACCGCCTTCTGTAG-3′) and *GAPDH* (forward, 5′-ACTTTGGTATCGTGGAAG GAC T-3′; reverse, 5′-GTAGAGGCAGGGATGATGTTC T-3′). Relative copy numbers (RCN) were calculated as 2−ΔCt, with ΔCt calculated by subtracting the average Ct of two housekeeping controls (*CAP1* and *GAPDH*) from the experimental sample Ct (Gavrilin et al., 2006; Butchar et al., 2008).

### ELISA

Cell-free supernatants were collected from resting and infected PBM and analyzed using sandwich ELISA kits specific for human TNF-α, IL-6, and IL-8 (R&D Systems). Each sample was tested in biological triplicates and instructions were followed according to manufacturer protocols.

### Immunostaining

Infected cells were fixed in 3.7% paraformaldehyde supplemented with 0.2% FBS for 30 min. Next, cells were washed two times with 1X PBS to remove residual paraformaldehyde. Cells were placed on poly-Lysine coated slides and allowed to adhere prior to blocking with a 5% FBS and 1% BSA solution in 1X PBS for 30 min. *F. novicida*-infected cells were incubated with primary anti *F. tularensis* subsp. *novicida* monoclonal antibody Fn8.2 (Immuno-Precise Antibodies Ltd; Victoria, British Columbia, Canada) at a 1:100 dilution for 4 h followed by the addition of Alexa Fluor 488 rabbit anti-goat antibody (Invitrogen, Carlsbad, CA). *F. tularensis* infected cells were incubated with primary mouse antibody raised against *F. tularensis* subsp *tularensis* LPS (Abcam, Cambridge, MA) at a 1:1000 dilution for 4 h followed by the addition of Alexa Fluor 594 goat anti-mouse antibody (Invitrogen, Carlsbad, CA). Cover slips were mounted onto the slides using VECTASHIELD mounting media. Images were captured using an Olympus BX41 fluorescent microscope equipped with a DP20 digital camera (Olympus) at 100X magnification. A minimum of 50 cells were analyzed per test group.

### Lactate dehydrogenase (LDH) cytotoxicity assay

LDH release from the cell was used as an indicator of cell death using an NAD^+^ reduction assay (Roche Applied Science). Supernatants from treated cells were collected, clarified by centrifugation at 400 g for 5 min, filtered and used for the assay. For a positive control, total LDH content in untreated monocytes was obtained by lysing cells with 1% Triton X-100. RPMI-1640 media was used as a blank and OD values were subtracted from readings of samples and positive control. LDH concentration in the medium was measured at 490 nm. Cell death was calculated by the formula: cytotoxicity (%) = [(sample-blank)/(positive control-blank) × 100], as described earlier (Gavrilin et al., 2012).

### Statistics

Student's *t*-test was used for comparison between two groups, and One-Way ANOVA was used for multiple group comparisons with a Tukey's Multiple Comparison *post-hoc* test to analyze significant differences. *p* ≤ 0.05 was considered to be significant. All experiments were performed a minimum of 4 independent times (*n* = 4) and results are expressed as mean values ± s.e.m.

## Results

### Virulent *F. tularensis* elicits a dampened cytokine response in human monocytes and suppresses responses to *F. novicida*

**Figure 1:**
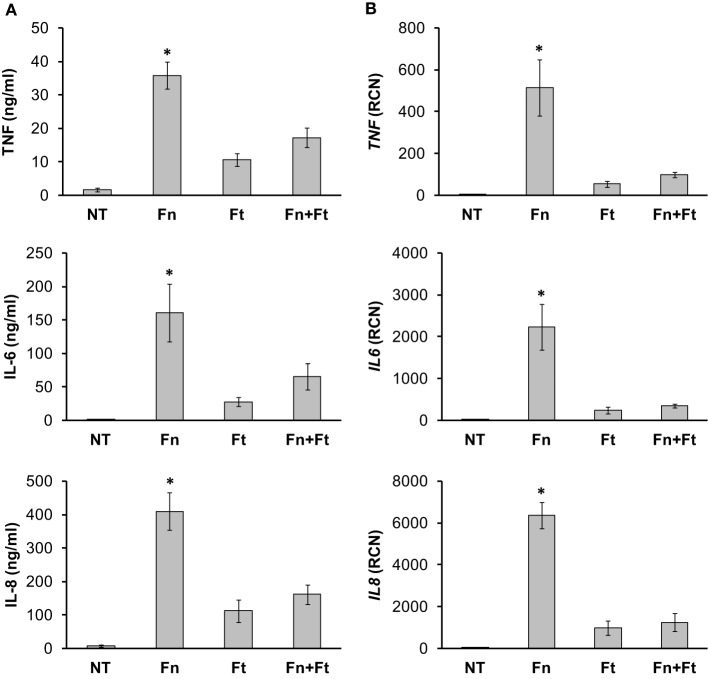
**Virulent strains of *F. tularensis* elicit a dampened cytokine response in monocytes**. Primary human monocytes were left untreated (NT) or infected in triplicate for 16 h with either *F. novicida* (Fn), *F. tularensis* Schu S4 (Ft), or both at an MOI of 50 for each bacteria. Cell-free supernatants from infected monocytes were collected and assayed by **(A)** sandwich ELISAs and **(B)** RT-qPCR for TNF-α, IL-6 and IL-8. “RCN” represents Relative Copy Number for the Y-axis. Graphs represent the mean ± s.e.m. from 4 independent donors. Data were analyzed by ANOVA. ^*^p < 0.01 (Fn vs. all groups). There was no significant difference between Ft and Fn + Ft.

It has previously been shown that *F. tularensis*-infected monocytes generate a limited pro-inflammatory cytokine response in contrast to those infected with *F. novicida* (Gavrilin et al., 2006, 2009; Butchar et al., 2008; Cremer et al., 2009), and that *F. tularensis* could lead to weaker responses to subsequent stimuli such as LPS (Bosio et al., 2007). Here, we aimed to determine the effects of *F. tularensis* on monocyte responses to *F. novicida* infection, with the expectation that active suppression (as opposed to evasion of detection) by *F. tularensis* would significantly dampen *F. novicida*-induced cytokine production. As a first step, we infected monocytes overnight with *F. novicida* (Fn) or *F. tularensis* SchuS4 (Ft) independently and measured cytokine responses by ELISAs and RT-qPCR. As expected, monocytes infected with *F. novicida* showed high production of TNF-α, IL-6 and IL-8 compared to those infected with *F. tularensis* SchuS4 (Ft) (Figure 1). After establishing the effects of these bacteria as single agents we proceeded to examine the effects of virulent *Francisella* on monocyte responses to the more pro-inflammatory *F. novicida*. To explore the possibility that active mechanisms are facilitating the observed immune suppression, we performed overnight infections of human monocytes with *F. novicida*, *F. tularensis* or both. Cell-free supernatants were collected, filtered, and assayed by ELISA for cytokine/chemokine production. Monocytes infected concurrently with *F. novicida* and *F. tularensis* displayed a dampened response similar to that seen with *F. tularensis* infections (Figure 1A), suggesting that *F. tularensis* was attenuating the response to *F. novicida*. We also assessed the cytokine mRNA levels induced after infection. Our results showed that *F. tularensis* infection led to significantly lower transcript levels both in single and mixed infections (Figure 1B). These results indicate that *F. tularensis* has a dominant immunosuppressive effect, as it was able to blunt the monocyte responses to the less virulent *F. novicida*.

### Bacterial interactions with monocytes do not differ between *F. novicida* and *F. tularensis*

**Figure 2:**
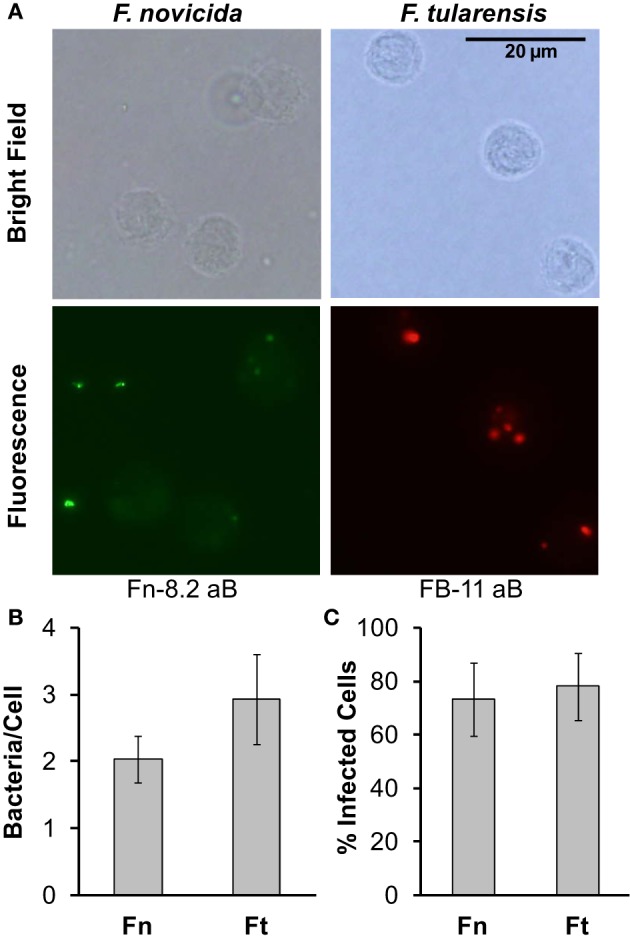
***F. novicida* and *F. tularensis* associate similarly with monocytes during infection**. Primary human monocytes were infected with *F. novicida* (Fn) or *F. tularensis* Schu S4 (Ft) at an MOI of 50 for 5 h. **(A)** Representative images from cells fixed in paraformaldehyde and then stained with Fn-82 antibody specific for *F. novicida* or FB-11 antibody specific for *F. tularensis*. The corresponding secondary antibody for Fn-82 was Alexa Fluor 488 rabbit anti-goat IgG (green) and for FB-11 it was AlexaFluor 594 goat anti-mouse IgG (red). Graphs represent the number of bacteria per cell **(B)** or the number of infected cells **(C)**. Graphs represent the mean ± s.e.m. from 1 donor incorporating a minimum of 4 frames. Data were analyzed by Student's *t*-test. No significant differences were found.

Since monocyte responses differ dramatically between *F. novicida* and *F. tularensis*, we aimed to determine if the effects of *F. tularensis* on monocyte responses compared to *F. novicida* infection were due to a difference in the number of bacteria associating with monocytes. To test this, we first infected monocytes for 5 h with *F. novicida* or *F. tularensis* at an MOI of 50. Following this, cells were washed two times, fixed in paraformaldehyde and stained with antibodies generated toward each specific bacterium as seen in representative images (Figure 2A). Our results show that although monocytes are associated with slightly lower numbers of *F. novicida* compared to *F. tularensis*, there is no statistically significant difference in the number of bacteria that associate with each cell (Figure 2B). The total percentage of cells associated with either *F. novicida* or *F. tularensis* is comparable, i.e., both bacteria associate with about 70% of the cells (Figure 2C).

### *F. tularensis* active suppression is dose-dependent

**Figure 3:**
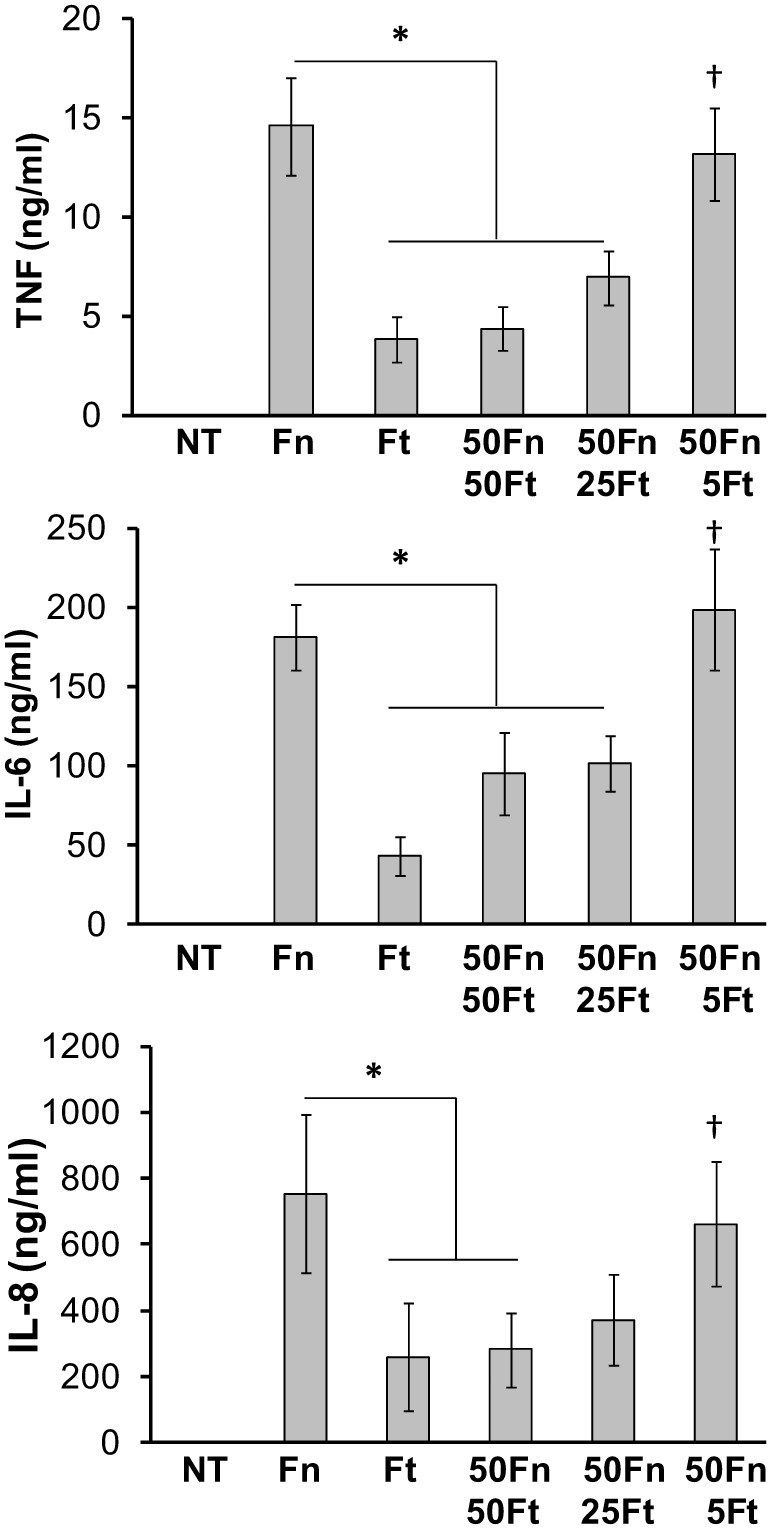
***F. tularensis* mediates immune suppression in a dose-dependent manner**. Primary human monocytes were left untreated (NT) or infected overnight (16 h) with *F. novicida* (Fn), *F. tularensis* (Ft), or a combination of the two. *Ft* MOI was administered at decreasing levels (50, 25, and 5 MOI) while keeping *Fn* MOI constant at 50. Cell-free supernatants were assayed by sandwich ELISAs for TNF-α, IL-6, and IL-8. Graphs represent the mean ± s.e.m. from 4 independent donors. Data were analyzed by ANOVA. ^*^p < 0.05 (Fn vs. selected groups), ^†^*p* < 0.01 compared to Ft.

Mixed-infection experiments described above were performed at a 1:1 ratio between *F. novicida* and *F. tularensis* to allow equal opportunity for both bacteria to evoke an immune response. Since *F. tularensis* was found to suppress the normal monocyte responses to *F. novicida*, we next asked whether a smaller ratio of *F. tularensis* to *F. novicida* could still lead to suppression. To test this we infected human monocytes with *F. tularensis* at an MOI of 50, 25, or 5 in conjunction with *F. novicida* at a constant MOI of 50. Our results indicated that the active suppression of *F. tularensis* was dose-dependent (Figure 3).

### Bacterial viability is necessary for *F. tularensis* mediated cytokine suppression

**Figure 4:**
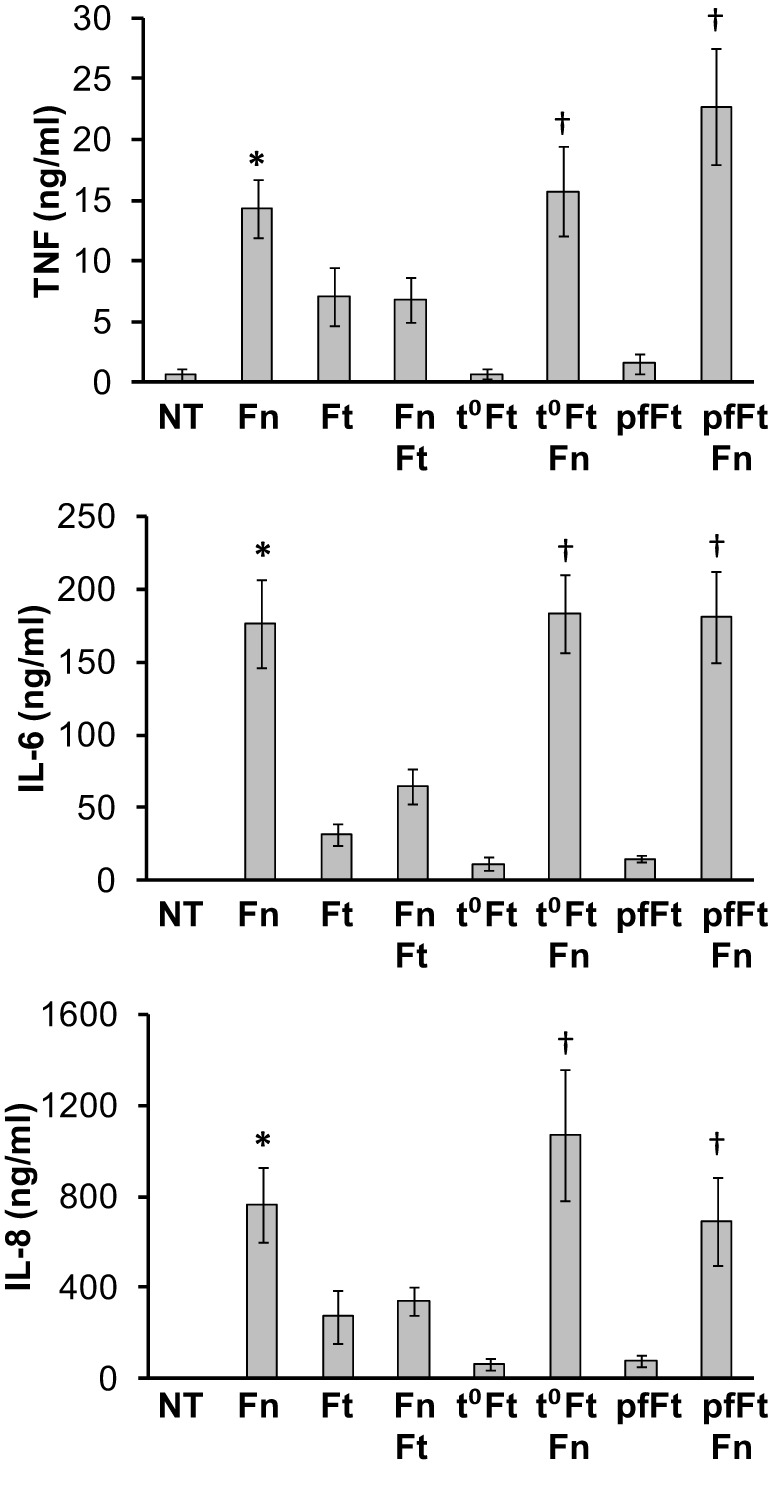
**Viability of *F. tularensis* is essential to mediate immune suppression**. Primary human monocytes were left untreated (NT) or infected overnight (16 h) with viable *F. novicida* (Fn), *F. tularensis* (Ft), or a combination of the two at an MOI of 50 for each bacteria. Additionally, monocytes were infected with heat-killed (t°Ft) or paraformaldehyde-fixed (pfFt) *F. tularensis* alone or in combination with live *F. novicida* (Fn). Cell-free supernatants from infected monocytes were assayed by sandwich ELISAs for TNF-α, IL-6, and IL-8. Graphs represent the mean ± s.e.m. from 4 independent donors. Data were analyzed by ANOVA. ^*^p < 0.05 (Fn vs. Ft and Fn + Ft); ^†^*p* < 0.05 compared to Ft, t°Ft, pfFt.

**Figure 5:**
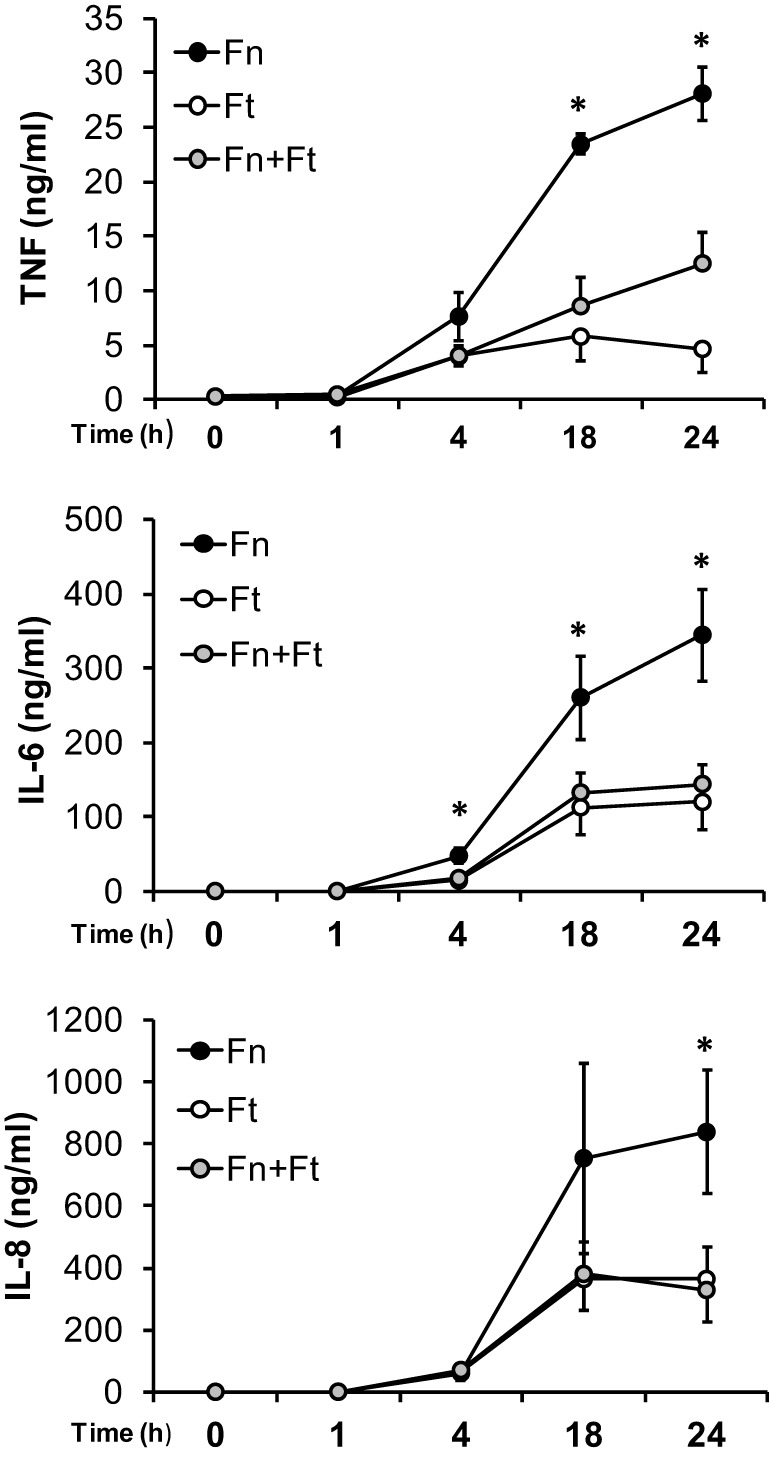
***F. tularensis* suppression is observed during early infection time points**. Primary human monocytes were infected at for 1, 4, 18, or 24 h with *F. novicida* (Fn), *F. tularensis* (Ft) or both at an MOI of 50 for each bacteria. Cell-free supernatants were collected and assayed using sandwich ELISAs for TNF-α, IL-6, and IL-8. Graphs represent the mean ± s.e.m. from 4 independent donors. Data were analyzed by ANOVA. ^*^p < 0.05 (Fn vs. Ft and Fn + Ft).

Having established that *F. tularensis* could actively suppress host cell cytokine responses in a dose-dependent manner, we next tested whether or not bacterial viability played a role. For this, monocytes were infected overnight with *F. novicida* along with live, paraformaldehyde-killed or heat-killed *F. tularensis*. Both paraformaldehyde and heat-killed *Francisella* were unable to induce a pro-inflammatory response from human monocytes (Figure 5). Monocytes co-infected with live *F. novicida* and killed *F. tularensis* showed cytokine responses similar to those infected with live *F. novicida* alone (Figure 4). These results are in agreement with Ireland et al. (2013), who found that bacterial viability was required for suppression of NF-κB and interferon responses. The requirement for viability suggests that rather than suppressing through contact alone, *F. tularensis* is producing and/or secreting one or more factors in order to effect the dampening.

### Time course of *F. tularensis*-mediated suppression

Previous *in vivo* studies reported that mice showed impaired pulmonary inflammatory responses to secondary stimuli when first challenged with *F. tularensis* (Bosio et al., 2007). These findings, combined with our above results showing that bacterial viability (and presumably production of immunosuppressive factors) was needed for the effects brought about by *F. tularensis*, led us to examine the length of time required for this suppression. Hence, we performed time course studies in monocytes infected with *F. novicida, F. tularensis* or both for 1, 4, 18, and 24 h. Supernatants were collected and analyzed by ELISA as above. Our results showed that although the greatest suppression occurred following overnight infection (18 and 24 h), co-infection with *F. tularensis* led to significant decreases in IL-6 cytokine suppression as early as 4 h after infection (Figure 5).

### *F. tularensis* can attenuate ongoing immune responses

**Figure 6:**
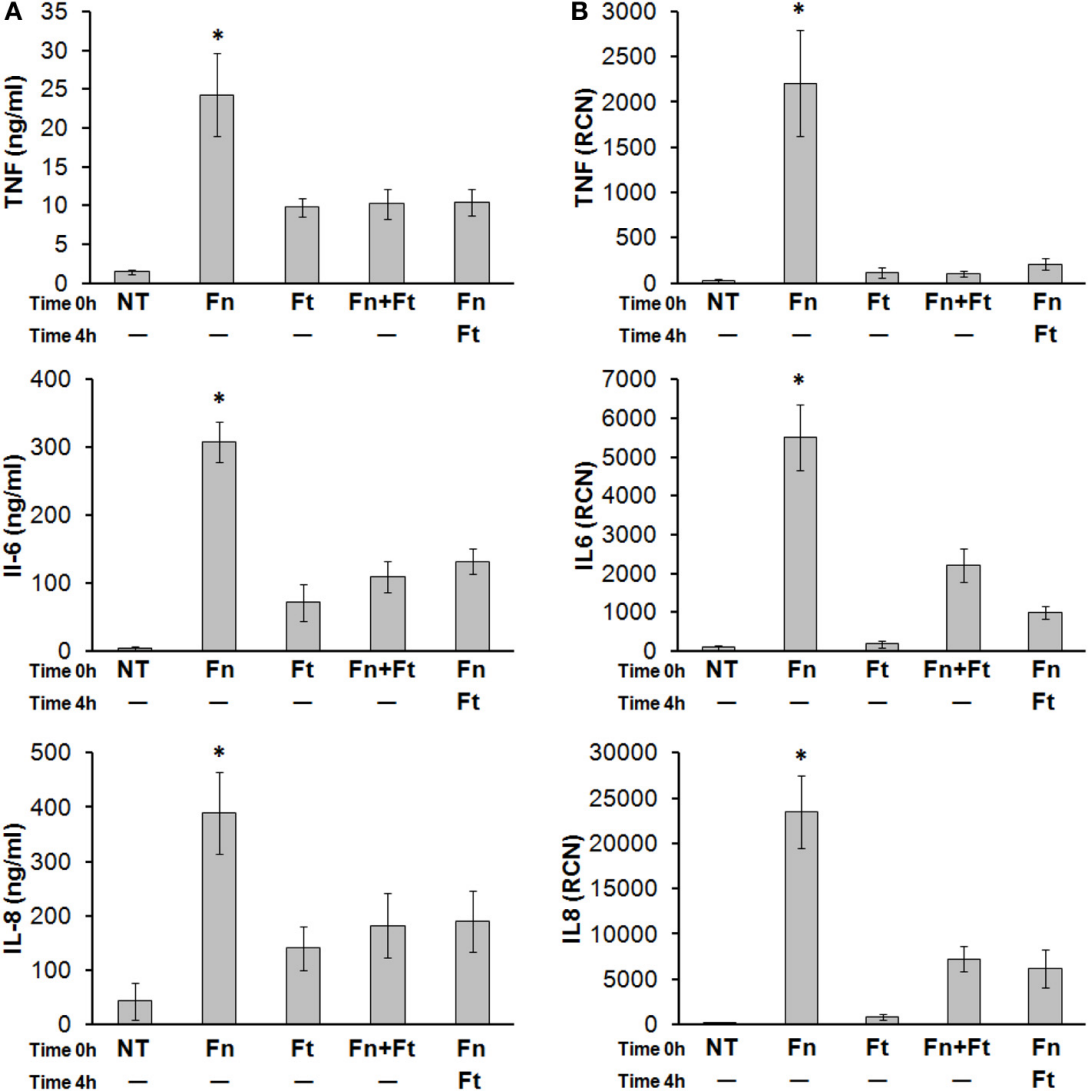
***F. tularensis* administered after *F. novicida* infection can still suppress the pro-inflammatory cytokine response elicited by *F. novicida***. Primary human monocytes were left untreated (NT) or infected overnight (16 h) with *F. novicida* (Fn), *F. tularensis* (Ft), or both at an MOI of 50 for each bacteria. In other samples, *F. tularensis* infection was performed 4 h following *F. novicida* infection. Cell-free supernatants were assayed by **(A)** sandwich ELISAs and **(B)** RT-qPCR, for TNF-α, IL-6, and IL-8. “RCN” represents Relative Copy Number for the Y-axis. Graphs represent the mean ± s.e.m. from 4 independent donors. Data were analyzed by ANOVA. ^*^p < 0.05 (Fn vs. all groups).

*F. tularensis* can begin dampening immune responses within just several hours of infection but its ability to inhibit an already-existing inflammatory response has not yet been demonstrated (Mares et al., 2008). To test this, we infected monocytes overnight with *F. novicida*, along with *F. tularensis* either concurrently or 4 h after *F. novicida*. Cells were lysed in TRIzol® and cleared supernatants were collected to measure cytokine transcript and secretion levels, respectively. Results showed that *F. tularensis* led to attenuated cytokine / chemokine responses even when added 4 h following *F. novicida* infection (Figures 6A,B). These results suggest that *F. tularensis* is likely interfering directly with one or more pro-inflammatory response pathways, as the bacteria are able to modulate responses already in progress.

## Discussion

Here, we provide evidence that *F. tularensis* can actively suppress host cell immune responses, including those already in progress. We chose to examine human peripheral blood monocytes, since *Francisella* predominantly targets these cells in the blood stream. Our results showed that human monocytes infected with *F. novicida* demonstrate robust pro-inflammatory responses. In contrast, co-infected with *F. tularensis* and *F. novicida* produced cytokines at low levels, similar to those seen with *F. tularensis* alone. Furthermore, *F. tularensis* was able to dampen monocyte responses even if administered several hours following infection with *F. novicida*. The cytokines TNF-α and IL-6 were reduced, and we also observed a significant reduction in the neutrophil-attracting chemokine IL-8 at 4 h after *F. tularensis* infection. This would lead to the prediction that neutrophil responses might be compromised at the whole-organism level after infection, but Hall *et al*. found a substantial neutrophil influx in mice infected with *F. tularensis* (Hall et al., 2008). Additional studies are needed to determine the degree to which the IL-8 reduction we observed would influence neutrophil responses *in vivo.*

*F. tularensis* has pleiotropic effects on individual cell types as well as whole organisms, and one of these is manipulation of cytokine profile. For example, Periasamy et al. (2011) showed that Th1 pro-inflammatory cytokines were absent within the first 72 h of pulmonary infection despite an intense neutrophil infiltrate and high bacterial burden. However, Th2 (Singh et al., 2013) and Th17 (Woolard et al., 2008) cytokines have been observed in mouse models of respiratory tularemia. It was postulated that the lack of Th1 pro-inflammatory response during the early phase of infection was mediated by such Th2 and Th17 cytokines (Periasamy et al., 2011).

Regarding our findings in human monocytes, it is unlikely that *F. tularensis*-induced shifts toward Th2 and Th17 responses explain its suppressive effect, either alone or during co-infection with *F. novicida*. For example, we found that *F. tularensis* SchuS4 supresses *IL10* gene expression in a pattern similar to Th1 cytokines (Supplementary Figure 1). In addition, *TGFB1* and *IL17RA* expression was suppressed in human monocytes by both *F. tularensis* and *F. novicida* (Supplementary Figure 1). In contrast to significant IL-17 response following respiratory *Francisella* LVS infection (Woolard et al., 2008), we were unable to detect reliable expression of *IL17A* in human monocytes infected with *Francisella* (data not shown), which is in agreement with the finding by Periasamy et al. in mouse lung macrophages (Periasamy et al., 2011). Thus, observed suppression of robust human monocyte pro-inflammatory responses for *F. novicida* by *F.tularensis* co-infection may not be explained by only Th2/Th17 activation as we did not detect this activation within the timeframe of our experiments. However, a Th2/Th17 response may be a potent regulator of the pro-inflammatory response during tularemia at the whole-organism level as other cells such as dendritic cells may contribute by releasing Th2 anti-inflammatory cytokines (Periasamy et al., 2011; Singh et al., 2013). Also, the difference in *Francisella* recognition between mice and men should not be ignored (Gavrilin and Wewers, 2011).

There is a possibility that host cell death rather than active suppression by *F. tularensis* is responsible for the reduced proinflammatory responses. Indeed, we observed this in the present study wherein infection by *F. tularensis* led to greater LDH release (Supplementary Figure2). Although cell death may contribute toward the observed suppression of cytokine production, reduced cytokine transcripts were also observed via RT-qPCR, which compares against 2 endogenous housekeeping genes (Figure 3). Moreover, we were able to observe differential increases in some genes such as *RELA* and *NFKBIA* following *F. tularensis* infection (Supplementary Figure 3).

Our results also showed that inhibition of monocyte cytokine production was dependent on the viability of *F. tularensis*, since heat- or paraformaldehyde-killed *F. tularensis* showed no effect. This is in agreement with the work by the Bosio group (Telepnev et al., 2003; Bosio and Dow, 2005; Bosio et al., 2007; Chase et al., 2009), who showed that live *Francisella* exposure could lead to an attenuation of responses to subsequent innate immune stimuli.

Numerous earlier studies have shown that *Francisella* is capable of evading host immune detection and eliciting suboptimal pro-inflammatory cytokine responses (Telepnev et al., 2003; Bosio and Dow, 2005; Andersson et al., 2006; Sjostedt, 2006; Chase and Bosio, 2010; Medina et al., 2010; Melillo et al., 2010; Zarrella et al., 2011). This phenomenon is not unique to *F. tularensis*, since other virulent pathogens such as the Ebola virus and *Mycobacterium leprae* show similar characteristics (Bosio et al., 2003; Sinsimer et al., 2010). Further examinations of the mechanisms underlying host cell responses to such immunosuppressive pathogens will likely uncover additional commonalities that may ultimately lead to new host-directed therapeutic strategies.

It has often been suggested that *Francisella* could, at least to some degree, directly antagonize pro-inflammatory responses (Metzger et al., 2007) as well as escape detection. Discerning between the two possibilities has been problematic, and further complicated by the possibility that *F. tularensis*-mediated suppression of immune responses could be a reflection of endotoxin tolerance, wherein immune cells become refractory (early phase) and desensitized (later phase) to immune stimuli following exposure to an initial stimulus such as mycobacterium, LPS or TNF-α (Greisman and Hornick, 1975; West and Heagy, 2002; Bosio et al., 2007; Morris and Li, 2012; Dai et al., 2013). However, recent work (Bosio et al., 2007; Dai et al., 2013) along with work detailed in this study provides strong evidence that a component of active suppression exists. Also, the dependence of suppression on *F. tularensis* viability in this study suggests that *F. tularensis* is producing one or more immunosuppressive agents that act on the host cell and that this effect is a dose-dependent manner. Alternatively we cannot rule out that the lower MOIs of *F. tularensis* resulted in the infection of fewer monocytes with this bacterium (vs. *F. novicida* at 50 MOI presumably infecting a greater number of monocytes).

Multiple mechanisms have been discovered to date by which *Francisella* defeats host immune responses (Bosio et al., 2007; Cremer et al., 2011; Jones et al., 2012). These include an unconventional LPS that is poorly recognized by TLR4 (Duenas et al., 2006; Bosio, 2011), surface lipopeptides such as Tul4 that induce TLR2 signaling but fail to elicit a strong cytokine response (Thakran et al., 2008), OmpA that prevents nuclear translocation of NF-κB p65 (Mahawar et al., 2012), the pathogen's interactions with CR3 (Balagopal et al., 2006; Ben Nasr et al., 2006; Ben Nasr and Klimpel, 2008; Barker et al., 2009; Dai et al., 2013), and its ability to avoid both serum-mediated killing and antibody detection (Bosio et al., 2007; Ben Nasr and Klimpel, 2008; Clay et al., 2008). *F. tularensis* also leads to host cell transcriptional changes such that immune response pathways such as IFNγ, PI3K, Erk and TLR2 may be weakened (Butchar et al., 2008). Earlier results from our microarray study showed that expression of the Akt-inactivating phosphatase PTEN was higher in monocytes infected with Schu S4 than with *F. novicida* (Butchar et al., 2008), and it has been shown in human monocyte-derived macrophages (MDM) that Schu S4 induces higher levels of PTEN (Melillo et al., 2010). Interestingly, as well as inducing increased PTEN levels, *F. tularensis* Schu S4 also inhibited the inactivation of PTEN in human MDM via antioxidant activity, again leading to dampened Akt phosphorylation during infection (Melillo et al., 2010). Our group also confirmed that the Akt antagonist, PTEN, is induced in Schu S4 infections. Collectively, these findings point out the multifaceted nature of *Francisella* with regard to overcoming immune responses and successfully infecting the host organism. As a facultative bacterium, *F. tularensis* possesses the ability to respond to changes in its immediate environment, which includes host cells and host organisms. Indeed, our group has shown that suppression can occur as early as during phagocytosis (Dai et al., 2013) and it has been shown that *F. tularensis* rapidly alters its own transcriptional profile during the course of host cell infection (Wehrly et al., 2009). As such, it is clear that *F. tularensis* employs a battery of methods in order to actively suppress host responses. Novel additional mechanisms will almost certainly be uncovered as genetic and biochemical studies become increasingly sophisticated

### Conflict of interest statement

The authors declare that the research was conducted in the absence of any commercial or financial relationships that could be construed as a potential conflict of interest.

## Supplementary material

Supplementary Figure 1 ***F. tularensis* suppresses expression of anti-inflammatory genes**. Primary human monocytes infected overnight (16 h) with *F. novicida* (Fn), *F. tularensis* (Ft), or both at an MOI of 50 for each bacteria were analyzed for expression levels of *IL10, TGFB1* and *IL17RA* genes. Data are expressed as mean ± s.e.m., *n* = 3 independent experiments.

Supplementary Figure 2 **LDH release from monocytes followed by infection with *Francisella***. Primary human monocytes were left untreated (NT) or infected for 8 h **(A)** or 18 h **(B)** with *F. novicida* (Fn), *F. tularensis* (Ft), or both at an MOI of 50 for each bacteria. Cell-free supernatants were assayed for LDH release as a signature of cell death. Data are expressed as mean ± s.e.m., *n* = 10 independent experiments.

Supplementary Figure 3 **Differential effect of *Francisella* on expression of selected genes in human monocytes**. Primary human monocytes infected overnight (16 h) with *F. novicida* (Fn), *F. tularensis* (Ft), or both at an MOI of 50 for each bacteria were analyzed for expression levels of *RELA*, *IKBIA, PSTPIP1* and *PYCARD* genes. Data are expressed as mean ± s.e.m., *n* = 3 independent experiments.
