# Supplementary figures and images for: Virulent Type A Francisella tularensis actively suppresses cytokine responses in human monocytes
Source: Front Cell Infect Microbiol. 2014 Apr 10;4:45. doi: 10.3389/fcimb.2014.00045 (PMC3988375; doi:10.3389/fcimb.2014.00045)

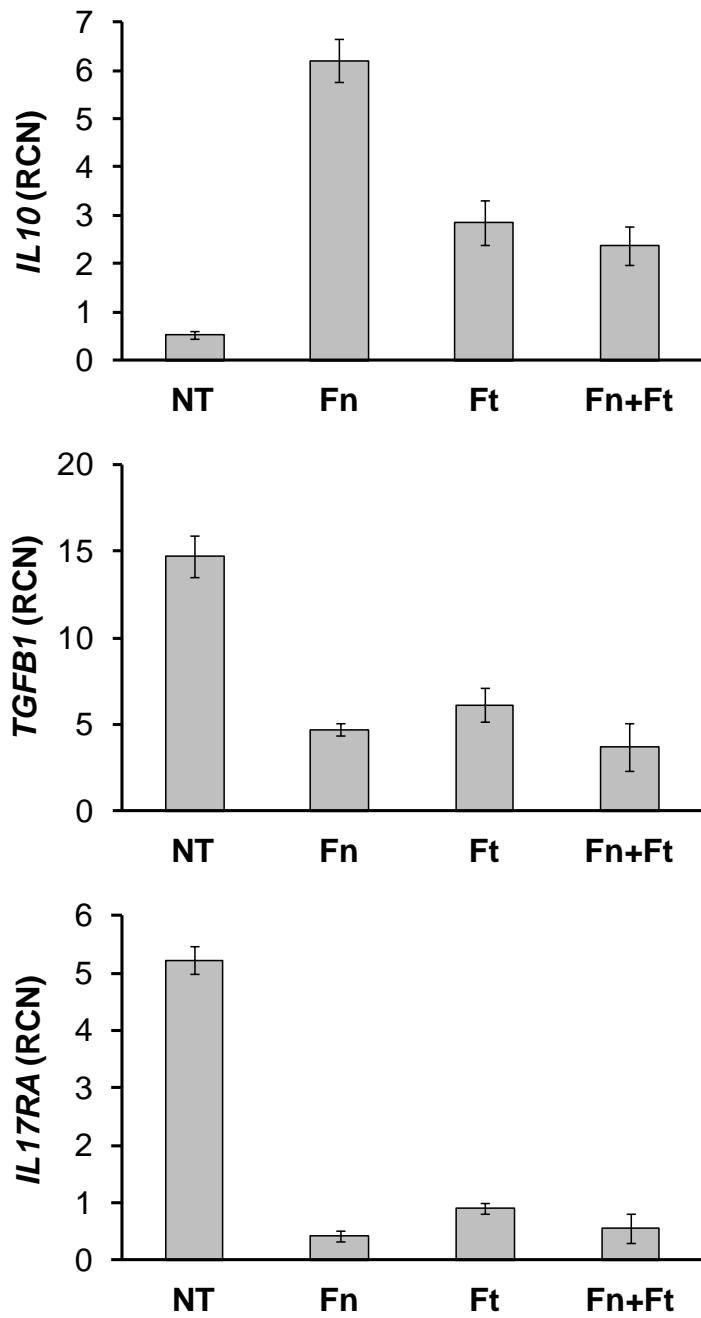

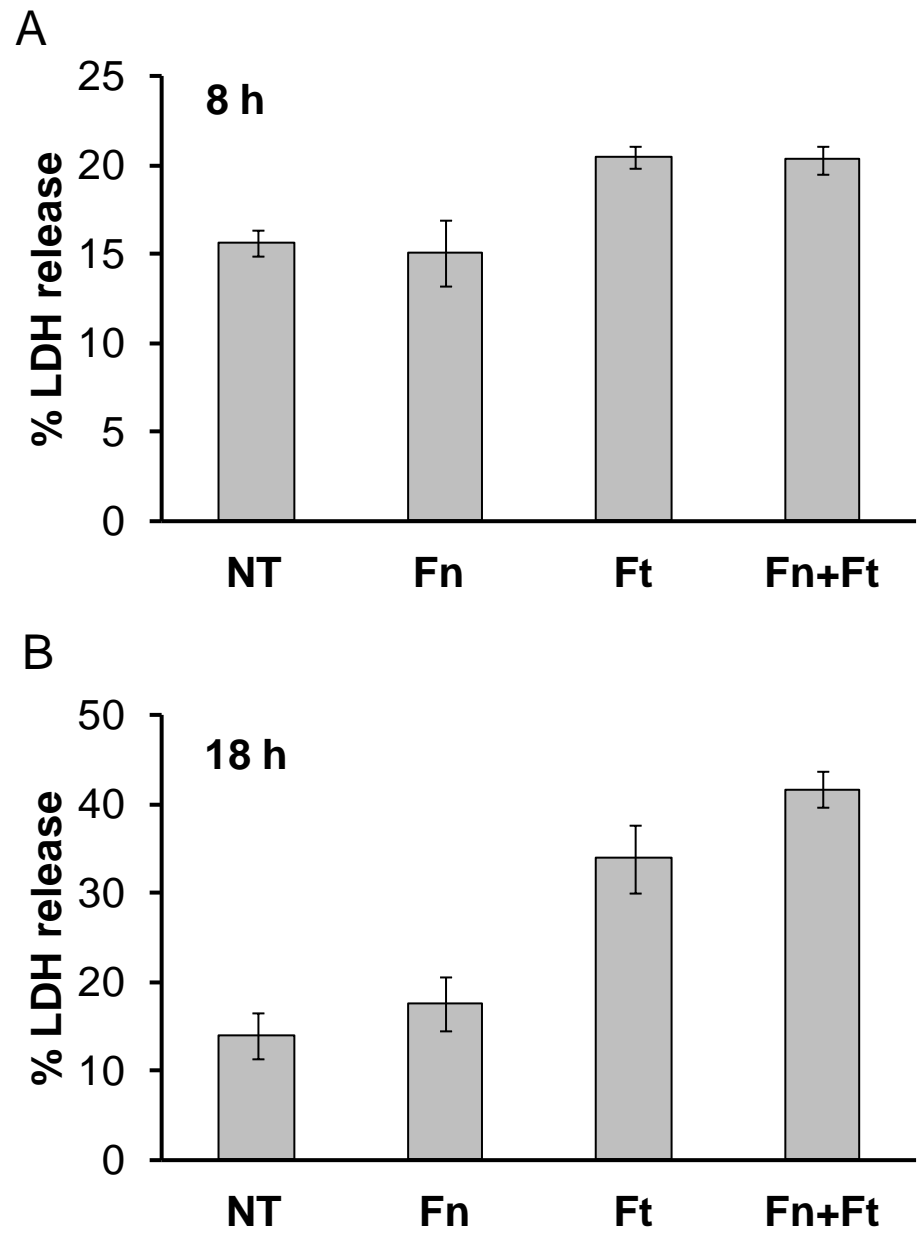

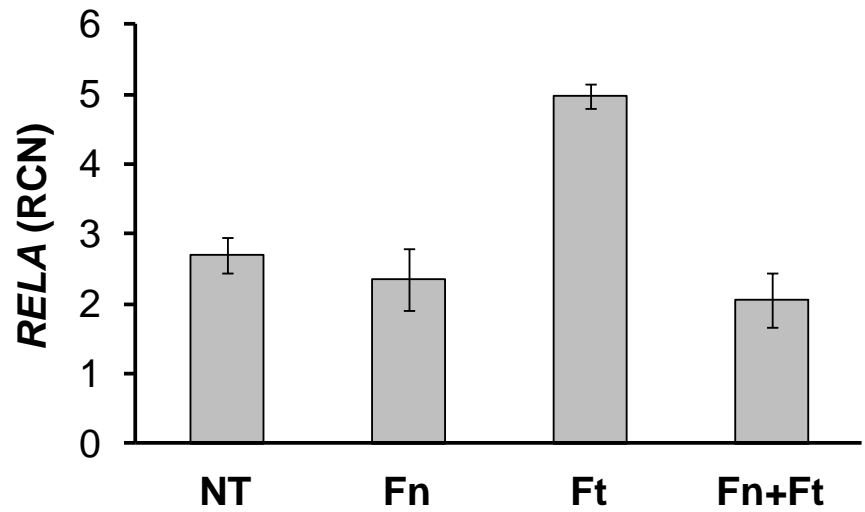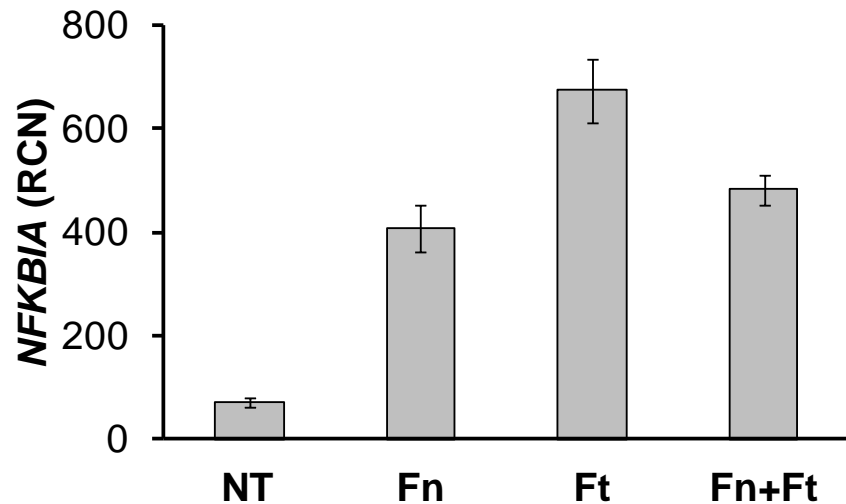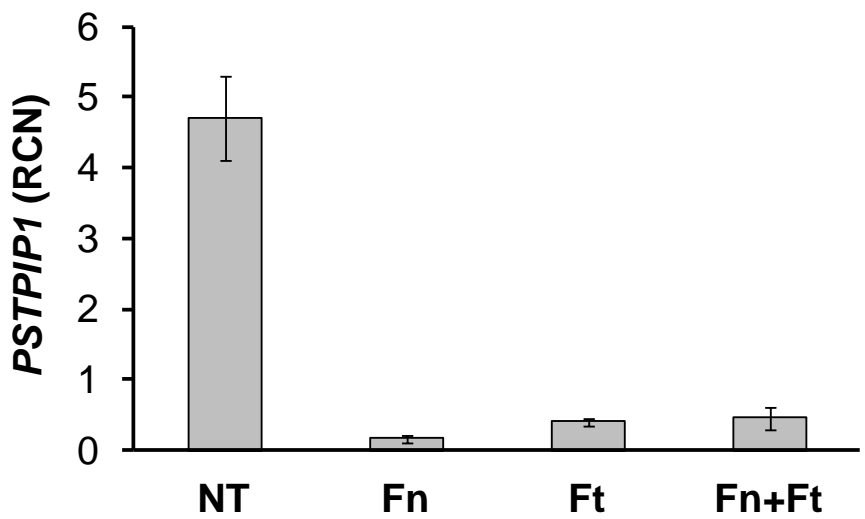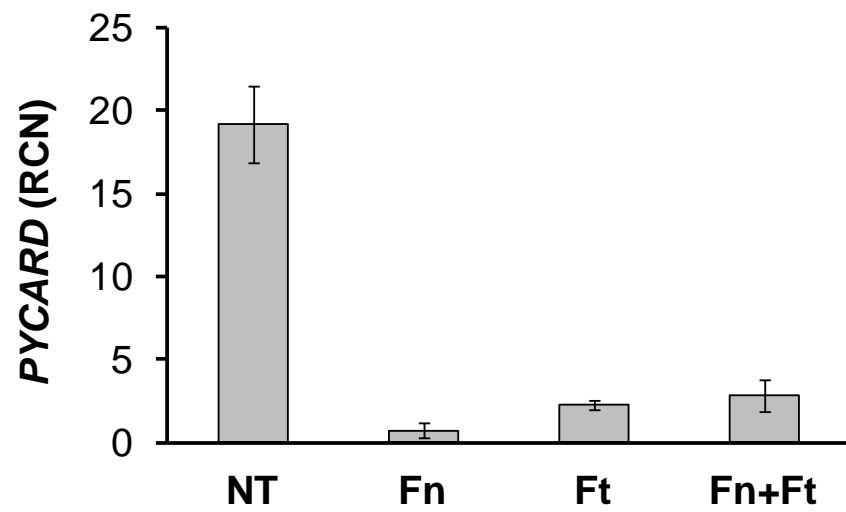

Supplement: Supplementary Figure 1 — F. tularensis suppresses expression of anti-inflammatory genes. Primary human monocytes infected overnight (16 h) with F. novicida (Fn), F. tularensis (Ft), or both at an MOI of 50 for each bacteria were analyzed for expression levels of IL10, TGFB1 and IL17RA genes. Data are expressed as mean ± s.e.m., n = 3 independent experiments. [file Presentation1.PDF]
